# The Combination of Retinal Neurovascular Unit Changes With Carotid Artery Stenosis Enhances the Prediction of Ischemic Stroke

**DOI:** 10.1167/tvst.14.3.14

**Published:** 2025-03-13

**Authors:** Zhifan Chen, Shuoxin Liao, Guangzhong Chen, Changmao Li, Chunling Liu, Junbin Liu, Guangyu Wu, Zheng Lyu, Mengya Liu, Xiyu Wu, Guixian Ma, Qianli Meng

**Affiliations:** 1 Department of Ophthalmology, Guangdong Eye Institute, Guangdong Provincial People's Hospital (Guangdong Academy of Medical Sciences), Southern Medical University, Guangzhou, China; 2 The Fourth Affiliated Hospital of Guangzhou Medical University, Guangzhou, China; 3 Department of Neurosurgery, Guangdong Provincial People's Hospital (Guangdong Academy of Medical Sciences), Southern Medical University, Guangzhou, China; 4 Department of Neurology, Guangdong Neuroscience Institute, Guangdong Provincial People's Hospital (Guangdong Academy of Medical Sciences), Southern Medical University, Guangzhou, China; 5 Department of Radiology, Guangdong Provincial People's Hospital (Guangdong Academy of Medical Sciences), Southern Medical University, Guangzhou, China

**Keywords:** ischemic stroke (IS), carotid artery stenosis, retinal neurovascular unit (RNVU), full-field electroretinography (FERG)

## Abstract

**Purpose:**

We aimed to analyze retinal neurovascular unit (RNVU) alterations and function via optical coherence tomography angiography (OCTA) and full-field electroretinography (ERG) in patients with ischemic stroke (IS).

**Methods:**

OCTA was used to measure RNVU changes in 229 participants (101 with IS and 128 healthy controls). The RETeval device was used to record full-field electroretinograms (FERGs) in 40 participants (14 with IS and 26 healthy controls). Logistic regression models for IS were constructed. Receiver operating characteristic (ROS) curves were constructed to assess the predictive value of various models for IS.

**Results:**

Patients with ipsilateral internal carotid artery stenosis (ICAS) had a greater occurrence of IS. A decrease in the vascular density (VD) of the parafovea, FD-300, and nasal optic disc; a decrease in the thickness of the retinal nerve fiber layer (RNFL) around the nasal optic disc; and an increase in the acircularity index (AI) were observed in patients with IS (*P* < 0.05). An increase in the AI was identified as a risk factor for IS, whereas the other factors were found to be protective factors. The IS group presented a delayed a-wave implicit time and decreased b-wave amplitudes at the scotopic point. By incorporating traditional risk factors, the degree of ipsilateral ICAS, and OCTA parameters, a high predictive value for IS was achieved (area under the curve [AUC] = 0.933).

**Conclusions:**

Patients with IS without visible fundus lesions presented changes in the RNVU, characterized by reductions in retinal VD and RNFL thickness, alongside dysfunction of photoreceptor cells and bipolar cells. The combination of RNVU changes with traditional risk factors can enhance the prediction of IS, which provides valuable guidance for monitoring this disease.

**Translational Relevance:**

This study demonstrated that the combination of OCTA parameters, the degree of ipsilateral ICAS, and traditional risk factors could can enhance the prediction of IS. These findings provide valuable guidance for monitoring IS by assessing RNVUs.

## Introduction

Stroke is the second leading cause of death and the primary cause of long-term disability worldwide. Its incidence is increasing in developing countries, affecting 13.7 million people and causing the deaths of 5.5 million people annually.^1^ Stroke can be categorized into ischemic stroke (IS) and hemorrhagic stroke. IS refers to infarction of the brain, spinal cord, or retina, accounting for approximately 71% of all strokes worldwide^2^ and 60% to 80% in China.^3^ Most IS cases occur due to thromboembolism, with common sources of embolism being large-artery atherosclerosis, small-vessel disease (associated with elevated blood pressure and diabetes, which are particularly common in Asia), and atrial fibrillation.^4^ Patients with internal carotid artery stenosis (ICAS) or occlusion have an unstable arterial blood flow velocity, and atherosclerotic plaques in the carotid artery are prone to breakage and detachment, resulting in arterial occlusion, which is the leading cause of IS.

The gold standard for assessing the cerebral vasculature in stroke patients is still digital subtraction angiography (DSA), but this procedure is associated with hazards such as radiation exposure, contrast allergy, contrast nephrotoxicity, and anesthesia, in addition to being invasive.^5^ Despite the significant advancements in neuroimaging technologies, directly observing and assessing damage to the cerebral microcirculation in living organisms is still a challenging task. Interestingly, the retina shares a common embryonic origin with cerebral blood vessels and exhibits similar morphological and physiological characteristics. These similarities provide a unique opportunity to utilize the retina as a noninvasive and direct window for evaluating the microvasculature of the brain in vivo.^6^

Research findings indicate that abnormalities in the retinal microvascular morphology could reflect several pathophysiological changes associated with hypertension, hyperglycemia, inflammation, and hypoxia, thereby identifying the microvascular complications of diabetes^7^ and predicting the risks of cardiovascular disease, cerebrovascular disease, stroke, and stroke mortality.^8^^–^^10^ However, an imaging analysis based on fundus photographs has difficulty quantifying retinal microvascular alterations. Optical coherence tomography angiography (OCTA), a new technique that is rapid, noninvasive, and quantitative, offers a distinct advantage in the diagnosis, efficacy evaluation, and follow-up management of retinal choroid vascular changes and degenerative neuropathy.^11^ Its features make it ideal for evaluating and tracking retinal microvasculature changes in patients with stroke.^12^ Although recent studies have reported a reduction in retinal vessel density among individuals with IS,^13^^,^^14^ they failed to consider the status of ipsilateral ICAS or the implications for visual function.

Visual acuity is the most commonly utilized measure for assessing visual function. However, it is subjective and can be influenced by ocular media opacity. Thus, accurately reflecting the functional status of the retina becomes challenging. Electroretinography (ERG) is an objective, noninvasive, and sensitive method for detecting changes in retinal function. Full-field electroretinography (FERG) records the sum of the electrical responses of each layer of the retina, from photoreceptors to amacrine cells. However, traditional FERG devices require pupil dilation, corneal electrodes, and professional analyses, all of which significantly reduce device efficiency. The RETeval, a handheld ERG device, overcomes most of the shortcomings of traditional ERG devices and simplifies the evaluation of retinal function. The RETeval uses special skin electrodes to perform an FERG test quickly and noninvasively, eliminating the requirement for pupil dilation. It has been shown to be reliable and efficient in ERG studies of diabetic retinopathy (DR).^15^^,^^16^

The purpose of this study was to utilize OCTA and FERG to systematically observe and analyze the characteristic changes in the retinal microvasculature and neuronal structures, as well as retinal function, in patients with IS. Based on these results, we hope to further improve the accuracy of IS assessments by combining retinal neurovascular changes with ICAS.

## Methods

### Study Participants

Patients who were diagnosed with IS at Guangdong Provincial People’s Hospital from December 2019 to December 2021 were included in this study.

The inclusion criteria for the IS group were as follows: (1) aged 18 years and older; (2) confirmation of the diagnosis of unilateral IS (including large-artery atherosclerosis and small-vessel occlusion) through magnetic resonance imaging (MRI) or computed tomography (CT) findings; (3) ability to endure and cooperate during all ophthalmic examinations; and (3) no cardiogenic embolism, entrapment aneurysm, disorders of blood coagulation, erythrocyte true hypercellularity, vasculitis, or vascular malformation, which can cause IS.

The inclusion criteria for the control group were as follows: (1) aged 18 years and older; (2) no history of cerebral vascular disease, neurodegenerative illness, or any other condition affecting the central nervous system; and (3) recent imaging data such as B-ultrasound, CT, MRI, or DSA examination results revealing the internal carotid artery status, but no stroke occurred.

The exclusion criteria for both groups were as follows: (1) had additional severe systemic illnesses or central nervous system conditions that may affect the outcome of the tests; (2) had ocular diseases such as ametropia (≥ +3 diopters [D] or ≤ –3 D), choroidal and retinal diseases (including DR), optic neuritis, glaucoma, severe cataracts, intraocular inflammation, ocular trauma, or a history of intraocular surgery; and (3) poor-quality imaging findings.

A total of 229 patients with 229 eyes were ultimately included in this study, with 101 eyes in the IS group (the ipsilateral eye and internal carotid artery at the site of the IS) and 128 eyes in the healthy control group (the right internal carotid artery and the right eye). All participants signed a valid informed consent form. The research protocols of this study were authorized by the Medical Research Ethics Committee of Guangdong Provincial People’s Hospital (GDREC2020207H(R1)) and strictly followed the guidelines of the Declaration of Helsinki.

### Data Extraction

The demographic data included age, sex, history of hypertension, hyperlipidemia, diabetes, alcohol use, smoking status, and coronary artery disease. The laboratory test data included routine blood tests; coagulation function; and the levels of glycated hemoglobulin (HbA1c), total cholesterol (CHOL), triglyceride (TRIG), high-density lipoprotein (HDL), low-density lipoprotein (LDL), D-dimer (D-DI), and homocysteine.

The location of the IS was determined based on the clinical signs and the results of the auxiliary examination. Ipsilateral ICAS was noted based on the results of carotid imaging, with a priority order of DSA, CT, and B-mode ultrasound for examination. The degree of ICAS was divided into four grades according to the North American Symptomatic Carotid Endarterectomy Trial (NASCET): (1) mild stenosis < 50%; (2) moderate stenosis 50% to 69%; (3) severe stenosis 70% to 99%; and (4) complete occlusion > 99%.^17^

### Ophthalmic Examinations

All patients underwent a slit lamp examination combined with a 90-D lens, fundus photography, including macula-centered and optic disc-centered images, and OCTA (RTVue-XR Avanti; Optovue, Fremont, CA, USA). A FERG examination was performed on 40 of the 229 patients (14 with IS and 26 controls) using RETeval (LKC Tech. Inc., Gaithersburg, MD, USA; version 2.10.0).

The OCTA images were obtained using the split-spectrum amplitude decorrelation angiography algorithm incorporated with the RTVue-XR Avanti device with Angio Vue 2.0. The 6.0 mm HD Angio Retina centered on the fovea, the 4.5 mm HD Angio Disc scan centered on the disc, and ganglion cell complex (GCC) scan focused on the ganglion cells were utilized. The observed parameters, including the vessel density (VD; %) of the superficial capillary plexus (SCP) and deep capillary plexus (DCP), macular thickness, foveal avascular zone (FAZ), acircularity index (AI), retinal nerve fiber layer (RNFL) thickness, average GCC thickness, focal loss volume (FLV), and global loss volume (GLV), were described in detail in our previous article.^18^ This study only analyzes the results of images with a quality index > 6 points.

The FERG examination was performed using the special skin electrodes of the RETeval device and the nondilated pupil “ISCEV 6 step, light adapted first, Td” mode. In this mode, the RETeval device continuously measures the size of the pupil and adjusts the brightness of the flash to ensure consistent retinal illumination, regardless of the pupil size. This process is achieved using the following formula: Troland (Td) = (pupil area in mm^2^) (luminance in cd/m^2^). The implicit time and amplitude of the scotopic and photopic electrical responses were recorded.

### Statistical Analysis

All the statistical analyses were conducted using SPSS Statistics 26 (IBM, Chicago, IL, USA). The Shapiro‒Wilk test was used to assess the normality of continuous variables. Normally distributed variables are presented as the means and standard deviations (mean ± SDs), and variables with non-normal distributions are presented as medians and interquartile ranges. The Independent sample *t*-test was performed if the two sets of data met the criteria for a normal distribution and chi-square test; otherwise, the Mann‒Whitney *U* test was used as a nonparametric test. The factor analysis was used to extract latent variables from OCTA parameters of various models, selecting principal components with initial eigenvalues > 1 and cumulative values > 80%. The OCTA parameters were categorized as follows: SCP VD, DCP VD, FAZ parameters, optic disc VD, and RNFL thickness. The univariate regression analysis was used to examine the latent variables that were significantly different between groups. Latent variables in each category and baseline parameters with significant differences were included in the multivariate binary regression model to explore the risk factors for IS.

A receiver operating characteristic (ROC) curve was constructed to assess the predictive value of various models for IS, and the area under the curve (AUC) was calculated. The optimum cutoff value was established using the highest Youden index (sensitivity + specificity-1), and the corresponding sensitivity and specificity were recorded. The *P* values are reported as outcomes, with *P* < 0.05 considered statistically significant.

## Results

Compared with individuals in the healthy control group, patients in the IS group presented several distinguishing factors, including an older age; a greater proportion of patients with hypertension, hyperlipidemia and a smoking history; and a greater incidence of ipsilateral ICAS, with a greater degree of severity (*P* < 0.05). A significant difference in the incidence of diabetes was not observed between the two groups. In terms of laboratory results, patients with IS had lower HDL levels than did those in the healthy control group. Although no significant differences in white blood cell counts were observed between the two groups, patients with IS had higher neutrophil ratios and lower lymphocyte ratios. The demographic and clinical characteristics of both groups are illustrated in Supplementary Table S1.

Table 1 presents a comparison of macular VD between the IS group and the healthy control group. Compared with the healthy control group, the IS group presented a decrease in SCP VD in the parafoveal region. The overall DCP VD in the macular region was significantly decreased in the IS group compared to the healthy control group, except for the fovea. This difference was observed not only in the parafoveal region but also in the perifoveal region. However, no significant difference in macular retinal thickness was observed between the two groups. Compared with that in the control group, the AI in the IS group was significantly greater, whereas FD-300 was significantly lower. No significant differences in the FAZ area and perimeter (PERIM) were observed between the two groups.

**Table 1.** Comparison of Macular Vessel Density Parameters Between the Ischemic Stroke and Healthy Control Groups

	SCP			DCP		
Macular VD (%)	IS	Control	*P* Value	IS	Control	*P* Value
Whole image	46.27 ± 7.09	48.34 ± 4.18	0.010^*^	46.07 ± 7.14	48.41 ± 5.82	0.007^*^
S-Hemi	46.42 ± 7.78	48.82 ± 4.18	0.006^*^	46.25 ± 7.83	49.33 ± 5.77	<0.001^*^
I-Hemi	46.12 ± 6.67	47.84 ± 4.40	0.027^*^	45.91 ± 6.72	47.50 ± 6.07	0.062
Fovea	17.42 ± 7.73	17.62 ± 5.65	0.831	30.73 ± 8.85	32.67 ± 6.67	0.060
Parafovea	48.36 ± 5.86	50.15 ± 4.96	0.013^*^	50.93 ± 6.22	52.97 ± 4.65	0.005^*^
Para-S-Hemi	48.53 ± 6.83	50.86 ± 5.03	0.004^*^	50.93 ± 7.47	53.78 ± 4.53	<0.001^*^
Para-I-Hemi	48.19 ± 6.09	49.43 ± 5.34	0.105	50.94 ± 5.77	52.16 ± 5.24	0.096
Para-T	48.38 ± 6.87	50.19 ± 5.13	0.029^*^	51.58 ± 6.14	53.85 ± 5.04	0.002^*^
Para-S	49.25 ± 7.71	51.93 ± 5.26	0.003^*^	50.39 ± 8.45	53.49 ± 4.92	<0.001^*^
Para-N	47.45 ± 6.83	48.52 ± 5.94	0.206	51.98 ± 6.65	53.30 ± 4.78	0.081
Para-I	48.35 ± 6.55	49.93 ± 5.29	0.049^*^	49.80 ± 7.22	51.25 ± 5.64	0.089
Perifovea	47.76 ± 5.01	48.71 ± 4.21	0.121	46.96 ± 7.79	49.44 ± 6.48	0.009^*^
Peri-S-Hemi	48.04 ± 5.59	49.07 ± 4.11	0.125	47.47 ± 8.39	50.39 ± 6.36	0.004^*^
Peri-I-Hemi	47.43 ± 4.97	48.36 ± 4.58	0.144	46.51 ± 7.60	48.49 ± 6.89	0.040^*^
Peri-T	44.62 ± 5.86	44.87 ± 4.61	0.719	50.48 ± 8.68	52.24 ± 6.12	0.074
Peri-S	48.37 ± 5.66	49.37 ± 4.16	0.139	46.81 ± 8.78	49.66 ± 7.08	0.007^*^
Peri-N	51.11 ± 4.78	52.28 ± 4.44	0.057	46.22 ± 8.23	48.29 ± 6.90	0.039^*^
Peri-I	47.38 ± 5.24	48.30 ± 5.19	0.187	45.60 ± 7.65	47.57 ± 7.44	0.050
Parameters of FAZ						
FAZ area, mm^2^	0.33 ± 0.12	0.32 ± 0.10	0.541			
RERIM, mm	2.28 ± 0.45	2.19 ± 0.37	0.104			
Acircularity index, %	1.13 ± 0.06	1.10 ± 0.03	<0.001^*^			
FD-300, %	49.45 ± 7.23	52.08 ± 5.33	0.003^*^			

DCP, deep capillary plexus; FAZ, foveal avascular zone; FD-300, the VD within a 300-µm wide ring surrounding the FAZ; I, inferior; I-Hemi, inferior-Hemi; IS, ischemic stroke; N, nasal; RERIM, the FAZ perimeter; S, superior; SCP, superficial capillary plexus; S-Hemi, superior-Hemi; T, temporal; VD, vessel density.

* *P* < 0.05.

As shown in Table 2, the analysis of the optic disc capillary plexus revealed no significant difference in the overall VD of the optic disc between the two groups. However, the VDs of the inside optic disc, as well as those of the superior–nasal (SN), nasal–superior (NS), nasal–inferior (NI), and inferior–nasal (IN) regions of the optic disc, were significantly decreased in the IS group compared to the healthy control group. In the IS group, the RNFL thickness in the peripapillary, superior–temporal (ST), and SN regions of the optic disc area was significantly thinner than that in the healthy control group. No significant differences were observed in the GCC parameters between the two groups. Representative images of the vascular density and RNFL thickness in the two groups are shown in Supplementary Figure S1.

**Table 2.** Comparison of Optic Disc Vascular Density and Retinal Nerve Fiber Layer Thickness Parameters Between the Ischemic Stroke and Control Groups

	Optic Disc VD, %			RNFL Thickness, µm		
	IS	Control	*P* Value	IS	Control	*P* Value
Disc whole VD	53.81 ± 4.80	54.54 ± 5.56	0.306	–	–	–
Inside disc	58.15 ± 5.50	59.67 ± 3.93	0.021^*^	–	–	–
Peripapillary	55.61 ± 6.03	56.90 ± 3.27	0.058	111.57 ± 10.96	116.83 ± 11.94	<0.001^*^
TS	52.47 ± 5.39	53.08 ± 3.95	0.325	86.42 ± 34.91	82.09 ± 13.30	0.199
ST	51.83 ± 7.98	52.36 ± 4.62	0.553	132.33 ± 25.46	138.71 ± 20.95	0.040^*^
SN	47.75 ± 8.53	49.72 ± 4.61	0.040^*^	132.88 ± 26.38	141.30 ± 30.50	0.027^*^
NS	45.42 ± 5.83	49.62 ± 5.85	<0.001^*^	103.31 ± 16.54	106.63 ± 21.08	0.197
NI	43.91 ± 7.13	47.68 ± 5.53	<0.001^*^	83.98 ± 15.18	86.62 ± 16.00	0.209
IN	47.88 ± 8.49	51.12 ± 4.90	<0.001^*^	139.10 ± 34.63	143.79 ± 31.83	0.291
IT	54.59 ± 7.64	55.20 ± 4.81	0.462	144.69 ± 33.95	151.11 ± 27.42	0.116
TI	49.97 ± 6.37	50.03 ± 5.97	0.940	74.79 ± 18.25	74.88 ± 13.29	0.966

IN, inferior- nasal; IS, ischemic stroke; IT, inferior- temporal; NI, nasal - inferior; NS, nasal- superior; RNFL, retinal nerve fiber layer; SN, superior- nasal; ST, superior- temporal; TS, temporal- superior; TI, temporal- inferior; VD, vascular density.

* *P* < 0.05.

Table 3 displays the FERG waveform parameters of the IS group and the healthy control group. The results revealed that the IS group presented a delayed scotopic a-wave implicit time and decreased scotopic b-wave amplitudes at 85 Td*s (*P* < 0.05).

**Table 3.** Comparison of Full-Field Electroretinograms Parameters Between the Ischemic Stroke and the Healthy Control Groups

	Implicit Time, ms			Amplitude, µV		
	IS (*n* = 14)	Control (*n* = 26)	*P* Value	IS (*n* = 14)	Control (*n* = 26)	*P* Value
Scotopic						
DA0.28 b-wave	95.42 ± 17.00	92.87 ± 9.37	0.609	46.02 ± 20.97	55.75 ± 18.03	0.133
DA85 a-wave	18.50 (17.02, 19.70)	16.20 (15.30, 18.25)	0.036^*^	49.70 ± 12.18	51.14 ± 13.16	0.738
DA85 b-wave	46.83 ± 9.24	45.58 ± 4.73	0.643	72.17 ± 16.31	88.87 ± 19.34	0.009^*^
OPs	151.16 ± 15.03	148.31 ± 11.19	0.501	48.70 (37.70, 58.97)	57.65 (46.62, 72.77)	0.061
Photopic						
LA85 a-wave	12.45 (12.20, 13.30)	12.55 (11.75, 13.35)	0.670	8.02 ± 3.07	7.31 ± 2.28	0.405
LA85 b-wave	30.45 (29.22, 32.17)	30.15 (29.70, 31.95)	0.876	24.50 ± 5.40	24.21 ± 8.06	0.907
28.3 Hz flicker	26.20 (25.15, 27.90)	26.45 (25.90, 27.92)	0.268	24.12 ± 7.95	24.65 ± 8.62	0.853

DA0.28, scotopic b-wave to weak flashes at 0.28Td^*^s; DA85, scotopic a- and b-wave to strong flashes at 85Td^*^s; 28.3 Hz flicker, 28.3 Hz flicker ERG of the wave to repeated strong stimuli at 85Td^*^s; IS, ischemic stroke; LA85, photopic a- and b-wave to strong flashes at 85Td^*^s; OPs, the total oscillatory potential.

* *P* < 0.05.

Latent variables were extracted for the SCP, DCP, and optic disc parameters using a factor analysis. As shown in Supplementary Table S2, factors 1 through 3 correspond to the SCP VD in the parafoveal, perifoveal, and foveal regions, respectively; factors 4 and 5 correspond to the DCP VD in the parafoveal and perifoveal regions, respectively; factors 6 to 8 correspond to the VD in the nasal, temporal, and inside peripapillary areas, respectively; factors 9 and 11 correspond to the RNFL thickness in the temporal side of the optic disc; and factor 10 corresponds to the RNFL thickness in the nasal side of the optic disc.

Univariate logistic regression analysis revealed that participants with higher HDL levels had lower odds ratios (ORs = 0.335) of being diagnosed with IS, whereas those with a history of hypertension (OR = 2.626), hyperlipidemia (OR = 2.282), or smoking (OR = 1.887) had higher risks. For every degree of ipsilateral ICAS, a 1.721-fold greater risk of IS was observed. Participants with higher VDs of the SCP and DCP in the majority of the sectors, higher FD-300 values of the FAZ, and higher VDs of the optic disc were found to have lower likelihoods of being diagnosed with IS based on the characteristics observed with OCTA. In addition to traditional risk factors (TRFs), such as an older age, hypertension, hyperlipidemia, and smoking, a multivariate binary logistic regression analysis revealed that a decreased VD of the parafoveal disc, FD-300, and nasal optic disc; increased AI; and a thinner RNFL of the nasal optic disc were identified as risk factors for IS (Table 4).

**Table 4.** Risk Factors for Ischemic Stroke Using Binary Logistic Regression Analysis

Characteristics	Univariate Model OR (95% CI)	*P* Value	Multivariate Model OR (95% CI)	*P* Value
Baseline characteristics				
Age, y	1.028 (1.005–1.048)	0.017^*^	1.027 (1.002–1.052)	0.032^*^
History of hypertension	2.626 (1.493–4.621)	<0.001^*^	1.930 (1.020–3.651)	0.043^*^
History of hyperlipidemia	2.282 (1.203–4.328)	0.012^*^	2.263 (1.138–4.500)	0.020^*^
History of smoking	1.887 (1.067–3.339)	0.029^*^	2.562 (1.351–4.859)	0.004^*^
HDL, mmol/L	0.335 (0.129–0.874)	0.025^*^	N/A	0.132
N%	N/A	0.633	N/A	N/A
L%	N/A	0.272	N/A	N/A
Degree of ipsilateral ICAS	1.721 (1.420–2.085)	<0.001^*^	–	–
FAZ				
Acircularity index, (%)	1.328 (1.157–1.525)	<0.001^*^	1.298 (1.116–1.511)	0.001^*^
FD-300, (%)	0.934 (0.894–0.977)	0.003	0.857 (0.780–0.941)	0.001^*^
SCP VD				
Factor 1	0.667 (0.497–0.895)	0.007^*^	0.668 (0.498–0.896)	0.007^*^
Factor 2	N/A	0.598	N/A	0.651
Factor 3	N/A	0.313	N/A	0.300
DCP VD				
Factor 4	0.734 (0.552–0.977)	0.034^*^	0.736 (0.558–0.973)	0.031^*^
Factor 5	N/A	0.087	N/A	0.082
Optic disc VD				
Factor 6	0.515 (0.380–0.700)	<0.001^*^	0.502 (0.367–0.686)	<0.001^*^
Factor 7	N/A	0.276	N/A	0.191
Factor 8	N/A	0.062	N/A	0.052
Optic disc RNFL thickness				
Factor 9	N/A	0.092	N/A	0.081
Factor 10	0.627 (0.467–0.843)	0.002^*^	0.619 (0.458–0.837)	0.002^*^
Factor 11	N/A	0.646	N/A	0.677

DCP, deep capillary plexus; FAZ, foveal avascular zone; FD-300, the VD within a 300-µm wide ring surrounding the FAZ; HDL, high-density lipoprotein; ICAS, internal carotid artery stenosis; L%, lymphocyte ratio; N%, neutrophil ratio; RNFL, retinal nerve fiber layer; SCP, superficial capillary plexus; VD, vessel density.

* *P* < 0.05.

The AUC values of the TRF, degree of ipsilateral ICAS, and OCTA parameters were 0.739, 0.779, and 0.893, respectively. The model of OCTA parameters was constructed using latent variables, including factor 1, factor 4, factor 6, and factor 10. The AUC of the integrated model, which combined OCTA parameters, TRF, and the degree of ipsilateral ICAS, was 0.933, with a sensitivity of 0.921 and a specificity of 0.871 (see the Fig.).

**Figure.**
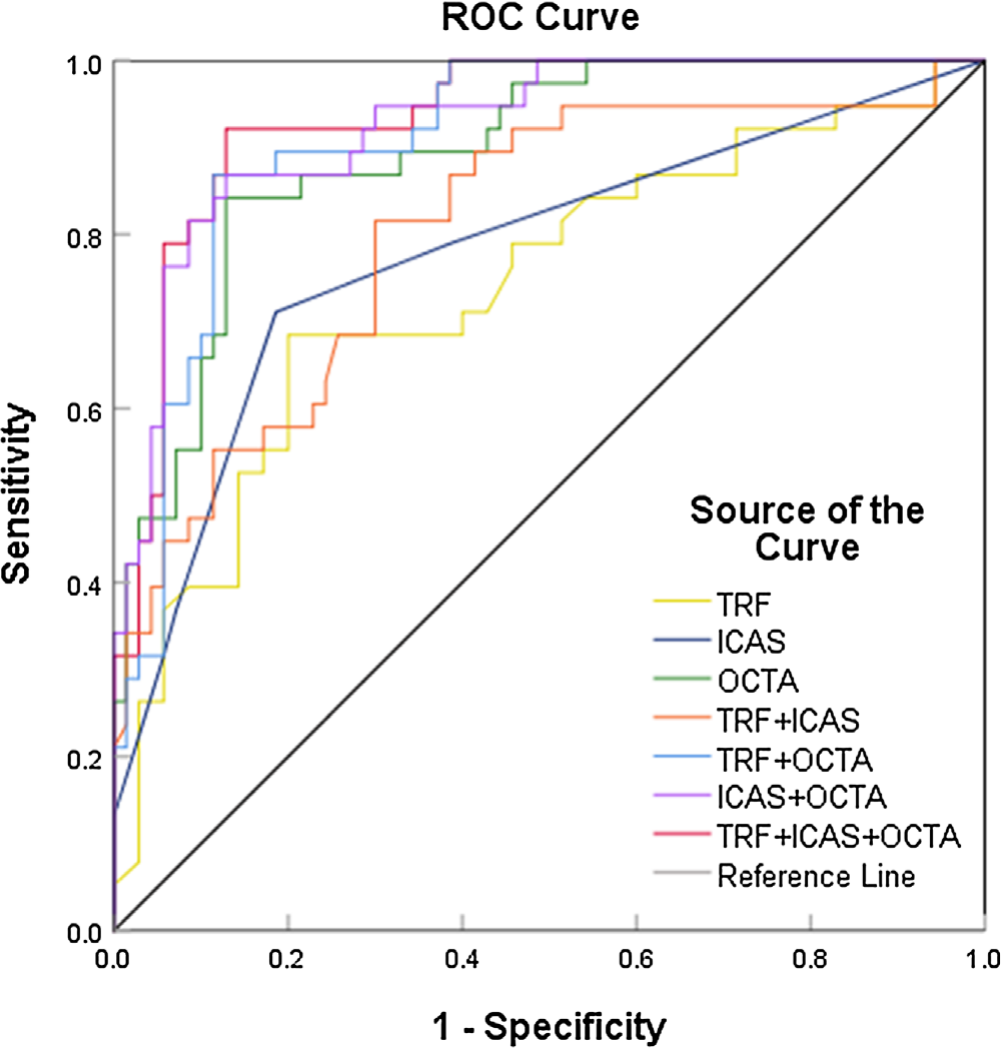
Diagnostic accuracy of different models in discriminating IS. The areas under the receiver operating characteristic curves (AUCs) of traditional risk factors (TRFs), ipsilateral ICAS and OCTA parameters (including FD-300, AI, and factors 1, 4, 6, 8, and 10) were 0.739, 0.779, and 0.893, respectively. The AUC for TRF + ICAS was 0.806; the AUC for TRF + OCTA was 0.910; the AUC for ICAS + OCTA was 0.923; and the AUC for TRF + ICAS + OCTA was 0.933, with a sensitivity of 0.921 and a specificity of 0.871.

## Discussion

In this study, we utilized OCTA to assess the parameters of the RNVU in the ipsilateral eye of patients with IS. We identified multiple risk variables for IS, including a decreased VD in the parafoveal area, FD-300, and nasal optic discs; an elevated AI; and a thinner RNFL in the nasal optic disc. By integrating traditional risk factors, the degree of ipsilateral ICAS, and OCTA parameters, the diagnostic accuracy of IS can be improved.

The neurovascular unit (NVU), introduced by the Stroke Progress Review Group,^19^ is a complex structural and functional unit that is essential for supporting the brain’s metabolic needs. Dysfunction of the NVU has been linked to several diseases, including stroke and Alzheimer's disease.^20^ In individuals with IS, the NVU is drastically disrupted, affecting both physical and molecular signaling and ultimately leading to blood‒brain barrier (BBB) damage. Because the brain (including the BBB) shares structural and functional similarities with the retina (including the blood‒retina barrier),^21^ the concept of the retinal neuronal vascular unit (RNVU) was proposed.^22^^,^^23^ Endothelial cells and neurons are critical components of the RNVU. Increasing evidence has shown that factors capable of inflicting damage to the cerebrovascular system can also result in injury to the retinal microvasculature and nerves.^24^^,^^25^

Our study revealed a reduction in the retinal microvascular density in patients with IS, including the VD of the SCP and DCP and the AI and FD-300 of the FAZ, which aligns with findings from recent studies.^13^^,^^14^ These findings suggest that the retinal microvasculature changed in these individuals prior to the development of noticeable fundus lesions. The microvascular metrics of OCTA in patients with certain diseases, such as DR,^26^ Parkinson's disease,^27^ and systemic lupus erythematosus,^28^ have been found to be correspondingly changed even in the absence of clinically visible pathological damage, suggesting the presence of capillary obstruction, hemodynamic disruption, or endothelial injury. Furthermore, these findings emphasize the high sensitivity of these parameters to ischemia and hypoxia.

We observed changes in neuron-related metrics. The results revealed that the RNFL of the optic disc, where nerve fibers cluster, was significantly thinner in patients with IS. The RNFL and ganglion cells reflect the circumstances of retinal axons, dendrites, and cell bodies in the body and play crucial roles in visual processing in the retina. Previous research using OCT revealed that a decrease in RNFL thickness is related not only to glaucoma but also to cerebral microvascular disease.^29^ This nonglaucomatous RNFL defect is usually caused by RNFL microinfarction, which is assumed to occur due to ganglion cell loss caused by local vascular insufficiency, and it acts as the basis for the transneuronal retrograde degeneration of retinal ganglion cells.^30^ Therefore, we speculate that the injury to retinal ganglion cells is caused by retinal hypoperfusion in patients with IS, and is manifested as a thinning of the optic disc RNFL.

In the IS group, a decreasing trend in the GCC and an increasing trend in the FLV and GLV were observed, but these values were not significantly different from those of the control group. Conversely, Liang et al.^13^ reported statistically significant differences in these parameters between the two groups. We hypothesize that the difference in the results between the two studies may be related to the sample size. In addition, our findings indicate that the retinal thickness in the macular region remains stable in patients with IS. Recent studies have documented that patients with Parkinson’s disease (PD) have a noticeable reduction in retinal thickness and volume in this area, which may be associated with a deficiency in dopaminergic ganglion cells and the apoptosis of dopaminergic-regulated ganglion cells.^31^ The difference may be attributed primarily to IS being caused by ischemia, whereas PD is characterized by neurodegeneration.

The FERG results showed that the IS group presented a prolonged implicit time for scotopic a-waves and reduced amplitudes of scotopic b-waves at 85 Td*s, which suggested that patients with IS might exhibit diminished functionality of photoreceptor cells and bipolar cells compared with the healthy control group. In a mouse model of unilateral common carotid artery occlusion-induced retinal ischemia, not only did cell necrosis and acute reactive gliosis occur but a decrease in retinal function was also observed in the early stage, as evidenced by significant decreases in the amplitudes of the a-wave, b-wave, and oscillatory potentials of electroretinograms.^32^ Patients who experience cerebral autosomal arteriopathy with subcortical infarcts and leukoencephalopathy (CADASIL) exhibit reduced amplitudes in FERG and pattern ERG, as well as delayed implicit times in pattern ERG and visual evoked potentials (VEPs).^33^ Studies of Alzheimer’s disease and vascular cognitive impairment have revealed that patients with dementia may exhibit ERG changes, such as decreased amplitudes and delayed peaks, even if they do not have a visual impairment.^34^^,^^35^ These changes could be caused by cerebral ischemia or retinal neurodegenerative alterations.^36^ However, to the best of our knowledge, no study has been conducted on the utilization of FERG in IS. Based on our findings, we hypothesize that retinal photoreceptor cells and bipolar cells are similarly affected when the retinal microvascular density and RNFL thickness are decreased in patients with IS.

Hypoxia and glucose deficiency due to ischemia may cause oxidative stress and induce the production of reactive oxygen species and inflammatory cytokines during IS.^37^ Neutrophils play a vital role as indicators of inflammatory responses, promote the release of proinflammatory cytokines, contribute to the formation of extracellular bactericidal networks, release proteases, interact with platelets, and promote thrombosis.^38^ Additionally, endogenous corticosteroid hormones are quickly released into the blood during the stress response, leading to the apoptosis of lymphocytes in peripheral blood and a subsequent decrease in the lymphocyte count. These findings explain the observations in our study that patients with IS have a higher neutrophil ratio and lower lymphocyte ratio than healthy control patients.

To the best of our knowledge, this study is the first to evaluate the occurrence of IS by analyzing retinal neurovascular features combined with ICAS. Moreover, this study is the first to assess the retinal function of patients with IS using the RETeval device. However, this study has several limitations. (1) Because this study is cross-sectional, inferring a causal association between the occurrence of IS and ICAS and changes in retinal microvessels and neurons is impossible. (2) Patients in this study had to have stable vital signs and be able to undergo eye examinations, which ultimately resulted in the inclusion of patients with comparatively mild IS. (3) Although the RETeval device and a standard ERG exhibit a high degree of agreement in the pediatric group, certain waveform differences have been observed in healthy adults.^39^ Additional evidence is needed to better understand the accuracy of the RETeval device in special populations, such as patients with IS.

In conclusion, this study revealed that in patients with IS, who do not have visible fundus lesions, alterations in the RNVU, marked by decreases in retinal VD and RNFL thickness, are accompanied by impairments in photoreceptor and bipolar cell function, which indicates that the RNVU parameters could serve as predictive biomarkers for IS. However, further large-scale prospective studies are necessary to include patients with IS of varying degrees, disease courses, and prognoses. These studies will help clarify the relationship between changes in the RNVU and the occurrence and progression of IS, thereby identifying the potential of alterations in the retinal structure and function for predicting IS.

## Supplementary Material

Supplement 1

Supplement 2
