# Supplementary material for: The Combination of Retinal Neurovascular Unit Changes With Carotid Artery Stenosis Enhances the Prediction of Ischemic Stroke
Source: Transl Vis Sci Technol. 2025 Mar 13;14(3):14. doi: 10.1167/tvst.14.3.14 (PMC11918090; doi:10.1167/tvst.14.3.14)
Supplement: Supplement 1 [file tvst-14-3-14_s001.pdf]

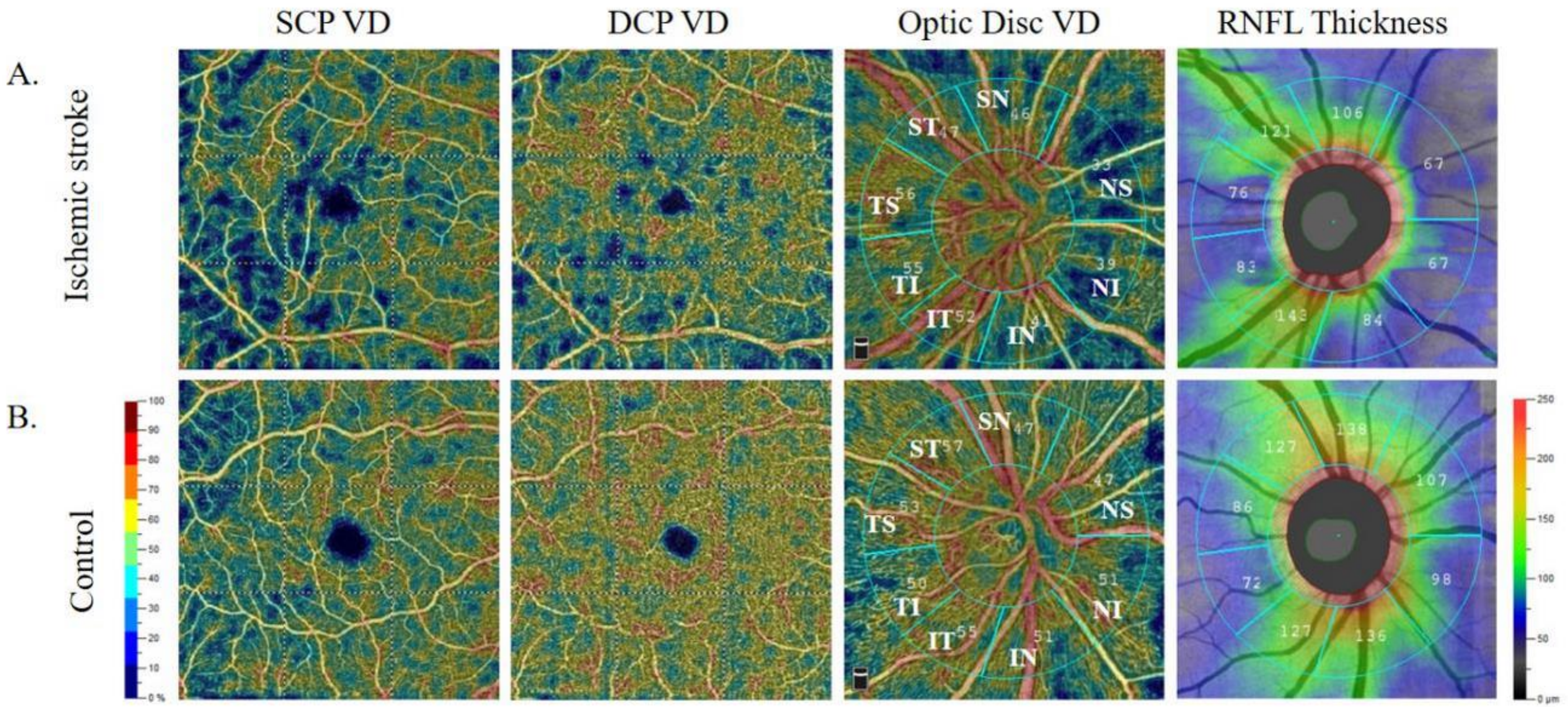

Supplementary Figure 1. Representative OCTA images of vascular density (VD) and retinal nerve fiber layer (RNFL) thickness of a 65-year-old male patient suffered from ischemic stroke along with right internal carotid artery stenosis exceeding 70% (A) and a 68-year-old male control (B). SCP, superficial capillary plexus; DCP, deep capillary plexus; NS,nasal superior; NI, nasal inferior; IN, inferior nasal; IT, inferior temporal; TI, temporal inferior; TS, temporal superior; ST, superior temporal; SN, superior nasal.
