# Supplementary material for: The Combination of Retinal Neurovascular Unit Changes With Carotid Artery Stenosis Enhances the Prediction of Ischemic Stroke
Source: Transl Vis Sci Technol. 2025 Mar 13;14(3):14. doi: 10.1167/tvst.14.3.14 (PMC11918090; doi:10.1167/tvst.14.3.14)
Supplement: Supplement 2 [file tvst-14-3-14_s002.pdf]

Supplementary Table 1. Comparison of baseline characteristics and parameters between the ischemic stroke and control groups.

Baseline Characteristics and Parameters	IS	Control	P - values
**Demographic Data**			
Patients	101	128	-
Sex, Male, n(%)	71(70.3)	90(70.3)	0.998
Age, (years)	63.33±12.18	59.04±13.20	0.012*
History of Hypertension, (%)	75(74.3)	67(52.3)	<0.001*
History of Hyperlipidemia, (%)	30(29.7)	20(15.6)	0.010*
History of Diabetes, (%)	38(37.6)	40(31.3)	0.312
History of CAS, (%)	25(24.8)	29(22.7)	0.711
History of Somking, (%)	38(37.6)	31(24.2)	0.028*
History of Alcohol Drinking, (%)	15(14.9)	12(9.4)	0.202
Degree of ipsilateral ICAS			<0.001*
None, (%)	39(38.6)	95(58.5)	
Mild, (%)	25(24.8)	19(19.2)	
Moderate, (%)	6(5.9)	3(3.9)	
Severe, (%)	18(17.8)	8(11.4)	
Occlusion, (%)	13(12.9)	3(7.0)	
**Laboratory parameters**			
CHOL, (mmol/L)	4.46±1.38	4.63±1.09	0.292
TRIG, (mmol/L)	1.85±1.66	1.53±0.77	0.057
HDL, (mmol/L)	1.06±0.25	1.15±0.31	0.023*
LDL, (mmol/L)	2.85±1.08	2.88±0.77	0.808
D-DI, (ng/mL)	720.63±320.77	610.00±533.07	0.456
HCY, (μmol/L)	13.30±4.50	12.43±5.79	0.231
UA, (μmol/L)	378.53±103.87	375.22±86.27	0.806
WBC, (10^9/L)	7.76±3.27	7.08±2.03	0.066
N, (%)	0.64±0.10	0.61±0.10	0.046*

L, (%)	0.26±0.10	0.31±0.23	0.039*
PLT, (10^9/L)	230.18±67.96	242.74±66.96	0.163
APTT, (s)	37.23±3.84	36.54±4.05	0.192
FIB, (ng/mL)	4.01±2.06	3.90±2.76	0.740
TT, (s)	18.28±12.46	16.22±0.81	0.100
PT, (s)	13.05±2.17	12.74±1.43	0.190

IS, ischemic stroke; CAS, coronary artery disease; ICAS, internal carotid artery stenosis; CHOL, cholesterol; TRIG, triglycerides; HDL, high-density lipoprotein; LDL, low-density lipoprotein; D-DI, d-dimmer; HCY, homocysteine; UA, uric acid; WBC, white blood cell; N%, neutrophil ratio; L%, lymphocyte ratio; PLT, platelet; APTT, activated partial thromboplastin time; FIB, fibrinogen; TT, thrombin time; PT, prothrombin time. *$P < 0.05$.

Supplementary Table 2. The latent variables for each category of optical coherence tomography angiography parameters using factor analysis.

Latent Variables	Parameters
SCP VD	
Factor 1	Whole Image, S-Hemi, I-Hemi, Parafovea, Para-S-Hemi, Para-I-Hemi, Para-T, Para-S, Para-N, Para-I
Factor 2	Perifovea, Peri-S-Hemi, Peri-I-Hemi, Peri-T, Peri-S, Peri-N, Peri-I
Factor 3	Fovea
DCP VD	
Factor 4	Parafovea, Para-S-Hemi, Para-I-Hemi, Para-T, Para-S, Para-N, Para-I
Factor 5	Whole Image, S-Hemi, I-Hemi, Fovea, Perifovea, Peri-S-Hemi, Peri-I-Hemi, Peri-T, Peri-S, Peri-N, Peri-I
Optic Disc VD	
Factor 6	SN, NS, NI, IN, Whole Image, Peri Disc
Factor 7	TS, ST, IT, TI
Factor 8	Inside Disc
Optic Disc RNFL Thickness	
Factor 9	ST, IT
Factor 10	SN, NS, NI, IN, Peripapillary
Factor 11	TS, TI

VD, vessel density; SCP, superficial capillary plexus; DCP, deep capillary plexus; S-Hemi, superior-Hemi; I-Hemi, inferior-Hemi; T, temporal; S, superior; N, nasal; I, inferior. TS, temporal-superior; ST, superior-temporal; SN, superior-nasal; NS, nasal- superior; NI, nasal-inferior; IN, inferior-nasal; IT, inferior-temporal; TI, temporal-inferior; FAZ, foveal avascular zone; RERIM, the FAZ perimeter; FD-300, the VD within a 300-μm wide ring surrounding the FAZ.
